# Effect of nursing application of emotion freedom technique on perceived stress, resilience and sexual satisfaction among women after mastectomy

**DOI:** 10.1186/s12912-025-02977-2

**Published:** 2025-04-15

**Authors:** Ashgan Fathy Mohamed, Alaa Eldin Moustafa Hamed, Samar Shaban AbdElazim Mohamed, Ahmed Abdellah Othman, Nahla Abdallah Abd El-Tawab

**Affiliations:** 1 Psychiatric and Mental Health Nursing Faculty of Nursing, Beni-Suef University, Beni-Suef, Egypt; 2 Psychiatric and Mental Health Nursing, Faculty of Nursing, Helwan University, Cairo, Egypt; 3 Maternity and Newborn Health Nursing, Faculty of Nursing, Beni-Suef University, Bani-Suif, Egypt; 4 Nursing Administration Faculty of Nursing, Sohag University, Sohag, Egypt

**Keywords:** Emotional freedom technique, Stress, Resilience, Sexual satisfaction, Mastectomy

## Abstract

**Background:**

A breast cancer diagnosis extends beyond physical health concerns, profoundly impacting a woman’s psychological well-being, resilience and sexual satisfaction. Mastectomy intensifies these challenges, potentially affecting overall quality of life and long-term well-being. Understanding the interrelationships between perceived stress, resilience and sexual satisfaction is crucial for designing targeted interventions that effectively support mastectomized women. This study evaluates the effectiveness of the Emotional Freedom Technique in mitigating stress, enhancing resilience and improving sexual well-being post-mastectomy.

**Methods:**

A quasi-experimental pretest-posttest design was employed with a convenient sample of 112 Egyptian women who had undergone mastectomy. Participants were recruited from the Outpatient Oncology Clinic at Beni-Suef University Hospital. Data were collected over six months (January–June 2024) and analyzed using SPSS version 26.0. The intervention consisted of six structured EFT sessions delivered over six weeks. Paired t-tests assessed pre- and post-intervention differences, while Pearson and Spearman correlation analyses examined relationships between variables to accommodate different data distributions.

**Results:**

Post-intervention assessments revealed statistically significant improvements. Perceived stress scores decreased from 32.42 ± 1.70 to 17.27 ± 2.96 (t = 49.130, *p* < 0.001, Cohen’s d = 3.2), resilience scores increased from 11.53 ± 1.67 to 31.46 ± 5.48 (t = 36.454, *p* < 0.001, Cohen’s d = 2.8) and sexual satisfaction scores improved from 17.03 ± 1.55 to 31.00 ± 4.31 (t = 13.245, *p* < 0.001, Cohen’s d = 2.5). Strong negative correlations were found between perceived stress and both resilience (*r* = -0.692, *p* < 0.001) and sexual satisfaction (*r* = -0.835, *p* < 0.001), while resilience and sexual satisfaction were strongly positively correlated (*r* = 0.890, *p* < 0.001).

**Conclusion:**

EFT is a cost-effective, non-invasive intervention that significantly reduces stress, enhances resilience and improves sexual satisfaction in women post-mastectomy. To optimize clinical integration, healthcare institutions should develop structured EFT training programs for nurses, incorporating theoretical foundations, hands-on practice and competency assessments. Standardized protocols should be established to guide EFT implementation in post-mastectomy care. Further research should explore long-term effects and broader applicability across diverse healthcare settings.

**Clinical trial number:**

NCT06583629 on 4/9/2024.

## Introduction

Breasts play a vital role in women’s lives, serving physiological, psychological and socio-cultural functions. Beyond their biological significance in lactation, breasts symbolize femininity, body image, sexual identity and social experiences. Consequently, any alteration in this aspect can lead to severe cognitive and emotional challenges. Women diagnosed with breast cancer often experience feelings of sadness, hopelessness, loneliness, discouragement and dissatisfaction. The clinical symptoms caused by the disease contribute to heightened perceived stress, reduced resilience and disrupted personal relationships, which can negatively affect their overall well-being [1].

Breast cancer remains a significant global health concern, ranking as the most frequently diagnosed cancer among women worldwide. According to the World Health Organization (WHO), approximately 2.3 million women were diagnosed with breast cancer in 2022, leading to 670,000 deaths globally [2, 3]. It accounts for 11.7% of all new cancer cases, making it the most prevalent malignancy among women and the second leading cause of cancer-related mortality [4]. In low-income countries, over 70% of cancer-related deaths occur, with breast cancer contributing to 15% of these fatalities among women. In Egypt, breast cancer represents 38.8% of all cancers affecting women, with an estimated 22,700 cases reported in 2020—a number projected to rise to 46,000 by 2050 [5, 6].

Delays in diagnosis and treatment significantly contribute to increased mortality rates, as survival outcomes are closely linked to early detection [7, 8]. Strengthening early screening programs and improving healthcare accessibility are essential for reducing the burden of breast cancer, particularly in resource-limited settings [9]. However, when diagnosed at an advanced stage, aggressive interventions such as mastectomy become necessary. While essential for disease management, mastectomy poses substantial physical, emotional, and psychological challenges.

Mastectomy, the surgical removal of one or both breasts, remains a standard treatment for breast cancer [10]. However, it often leads to significant psychological distress due to its impact on body image. In many cultures, breasts symbolize femininity, motherhood, and sexuality, making their loss emotionally distressing and associated with negative self-perception and impaired sexual satisfaction [11, 12]. This stress weakens resilience, impairs sexual well-being, and ultimately reduces overall quality of life [13, 14].

Mastectomy, the surgical removal of one or both breasts, remains a common treatment modality for breast cancer [10]. However, it often leads to significant psychological distress due to its impact on body image. In many cultures, breasts symbolize femininity, motherhood, and sexuality, making their loss emotionally distressing and associated with negative self-perception and impaired sexual satisfaction [11]. Additionally, mastectomy patients frequently experience heightened perceived stress defined as the appraisal of life events as unpredictable, uncontrollable, or overwhelming [12, 13]. This stress weakens resilience, impairs sexual well-being, and ultimately reduces overall quality of life [14–16].

In response to these challenges, interventions that effectively target both psychological distress and its associated consequences are essential. One such intervention is the Emotional Freedom Technique (EFT), a therapeutic approach that integrates cognitive and somatic elements to alleviate stress. EFT involves tapping on specific acupressure points while focusing on negative emotions or thoughts, helping to regulate stress responses, enhance resilience, and promote emotional well-being [17, 18]. Previous research has demonstrated its effectiveness in reducing anxiety, enhancing coping mechanisms and addressing psychological barriers to sexual satisfaction [19–21].

The interactions between perceived stress, resilience, and sexual satisfaction are complex and multidimensional. High perceived stress negatively affects sexual satisfaction by reducing libido and reinforcing negative body image [22]. However, resilience can moderate these effects by fostering adaptive coping mechanisms that help women manage stress and maintain a positive outlook on sexual health. For instance, resilient women are more likely to communicate openly with their partners, seek counseling, and engage in self-care practices that enhance their well-being and sexual satisfaction [23].

The Stress and Coping Transactional Model, developed by Lazarus and Folkman, explains perceived stress as an outcome of the interaction between individuals and their environment. According to this model, stress arises when individuals perceive environmental demands as exceeding their coping resources [24]. Cognitive appraisal and coping strategies play a critical role in mediating stress responses. EFT can be understood as a coping mechanism within this framework, as it aims to reduce negative thoughts and physiological arousal associated with stress, thereby lowering perceived stress levels [25].

Resilience Theory is essential for understanding how individuals navigate adversity. EFT contributes to resilience by enhancing emotional regulation and mitigating the impact of negative emotions. Regular practice of EFT enables women who have undergone mastectomy to develop stronger coping mechanisms, thereby fostering resilient behaviors [26]. Sexual health is a fundamental aspect of overall well-being, particularly for women post-mastectomy, as they experience significant changes in body image and self-esteem [27]. The Biopsychosocial Model provides a comprehensive framework for understanding sexual satisfaction by emphasizing the interplay of biological, psychological, and social factors. By reducing perceived stress and strengthening resilience, EFT can help women improve their sexual self-concept and satisfaction by alleviating physiological and psychological barriers [28].

Regardless of these promising findings, there is a significant lack of comprehensive studies examining the combined effects of EFT on perceived stress, resilience and sexual satisfaction particularly among women after mastectomy. Most existing research tends to focus on these outcomes separately or within different contexts, such as general anxiety reduction or trauma recovery, without specifically addressing the unique challenges faced by women who have undergone mastectomy [29]. Therefore, it is important to study the effect of nursing application of EFT on perceived stress, resilience and sexual satisfaction among women after mastectomy which can inform more effective interventions and support mechanisms, ultimately improving the quality of life for this population in addition help healthcare providers offer better, evidence-based care tailored to the unique needs of women recovering from mastectomy.

To address this gap, the study proposes the following hypotheses:

### H1

Nursing application of EFT significantly reduces perceived stress among women post-mastectomy.

### H2

Nursing application of EFT significantly enhances resilience among women post-mastectomy.

### H3

Nursing application of EFT significantly improves sexual satisfaction among women post-mastectomy.

### H4

There is a significant negative correlation between perceived stress and resilience among women post-mastectomy.

### H5

There is a significant negative correlation between perceived stress and sexual satisfaction among women post-mastectomy.

### H6

There is a significant positive correlation between resilience and sexual satisfaction among women post-mastectomy.

## Methods

### Aim of the study

This study set out to evaluate the effect of nursing application of emotion freedom technique on perceived stress, resilience and sexual satisfaction among women after mastectomy.

### Design and study setting

A quasi-experimental design was utilized for the study, which was conducted in the Outpatient Oncology Clinic at Beni-Suef University Hospital in Beni-Suef Governorate, Egypt. Beni-Suef Governorate is a predominantly rural region in Upper Egypt, with a population of approximately 3.2 million people. The hospital serves as a tertiary care center, providing specialized medical services to residents of Beni-Suef City and surrounding areas. The Outpatient Oncology Clinic is a dedicated facility within the hospital that offers comprehensive care to cancer patients during follow-up phases and postoperative oncology interventions. The clinic operates five days a week (Sunday through Thursday) from 9:00 AM to 2:00 PM and consists of two main rooms: a waiting room that accommodates approximately 20–25 patients and their families and an examination and care room used for medical assessments, consultations and treatment. The clinic is staffed by a multidisciplinary team, including two full-time oncologists, three registered nurses with specialized oncology training and administrative support personnel.

The clinic primarily serves patients from Beni-Suef City and nearby rural areas, treating various cancers such as breast, colorectal, liver and hematologic malignancies. Many patients face challenges related to healthcare access, including transportation barriers and financial constraints. Despite these obstacles, the clinic provides essential diagnostic tools, such as ultrasound machines and laboratory facilities for routine blood tests. Advanced imaging and specialized treatments, including radiation therapy, are also available. A key component of the clinic is its dedicated infusion center, specifically designed for the safe and efficient administration of chemotherapy.

This facility allows patients to receive treatment on an outpatient basis, eliminating the need for overnight hospitalization. Under the supervision of trained oncology nurses, eligible patients undergo chemotherapy in the infusion center, ensuring continuous monitoring and support throughout the process. The clinic’s patient-centered care emphasizes continuity and follow-up, with regular appointments to monitor treatment progress, manage side effects and assess overall well-being. Healthcare professionals provide education on symptom management, medication adherence and lifestyle modifications to improve patient outcomes. Despite operating in a resource-constrained setting, the clinic’s dedicated team and multidisciplinary approach play a critical role in delivering accessible cancer care to the rural population it serves.

## Participants

The required sample size was calculated using a priori power analysis with G*Power 3.1.9.7 [30]. Based on a small effect size of 0.36, a power of 98% and an alpha level of 0.05, a minimum sample of 112 participants was required. The researcher selected the point biserial model test for this analysis. Accordingly, 112 Egyptian women who had undergone mastectomy were recruited. To control confounding variables and ensure a homogenous sample, strict inclusion and exclusion criteria were applied.

**Fig. 1:**
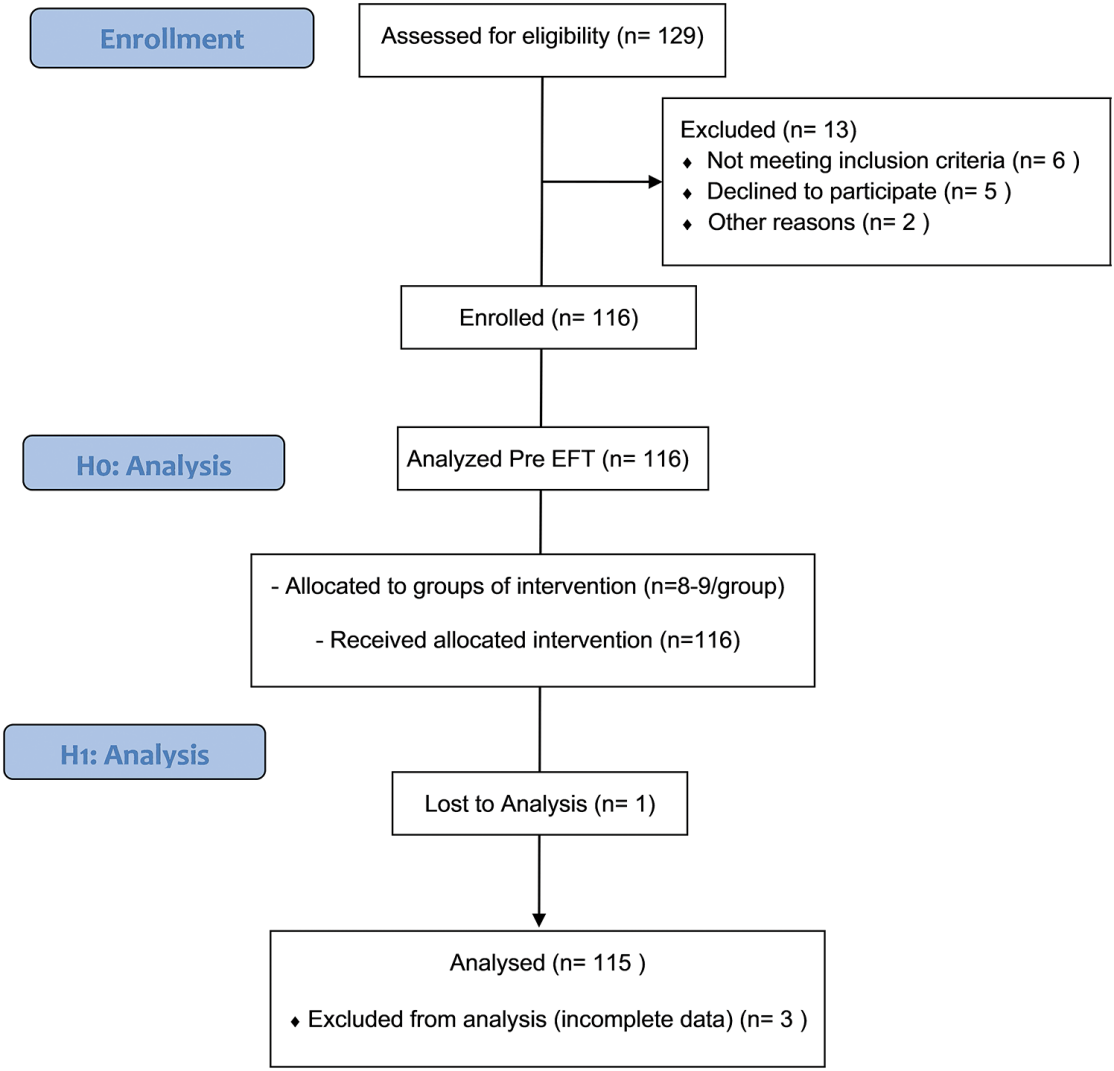
Participants enrollment diagram

Participants were eligible if they: (a) were within the first month post-mastectomy, (b) were married, (c) provided written informed consent and (d) had no prior exposure to emotional freedom techniques (EFT). Readiness to participate was determined by participants’ willingness to engage in all study activities, as confirmed during an initial screening interview. Exclusion criteria included: (a) a history of psychiatric disorders diagnosed by a healthcare professional, (b) chronic illnesses that could interfere with participation (e.g., uncontrolled diabetes or cardiovascular disease) and (c) any condition that prevented them from completing all study phases. Although this study did not include a control group, the intervention process was standardized to ensure consistency and minimize variability among participants. These measures were implemented to enhance the internal validity and reliability of the findings (Fig. 1).

### Blinding and potential biases

A limitation of this study was the lack of blinding, as participants were aware they were receiving an intervention. This awareness may have introduced potential biases, such as expectancy effects or social desirability bias, which could influence self-reported outcomes. Although blinding was not feasible due to the nature of the intervention, measures were taken to minimize bias. These included standardizing the intervention delivery, using structured assessment tools and ensuring data collection was conducted by research personnel who were not involved in the intervention process. These steps aimed to enhance the internal validity and reliability of the findings.

## Study instruments and measures

### Instruments of data collection

Four instruments were utilized to gather data for this study which comprised.

#### Instrument I: Participant’s *personal characteristics data sheet*

The researchers in the mother tongue “Arabic language” developed this structured sheet, containing the participant’s basic and educational information, including items such as: age, occupation, educational level, place of residence, economic status and medical history of the women as breast cancer duration, family member have breast cancer, mastectomy duration.

#### Instrument II Connor-Davidson Resilience Scale (CD-RISC)

The Connor-Davidson Resilience Scale (CD-RISC) was developed Kathryn and Jonathan, (2003) [31]. Connor-Davidson Resilience Scale (CD-RISC) comprises 25 items that measure resilience or capacity to change and cope with adversity. Respondents indicated their response on a 5-point Likert scale with higher scores indicating greater resilience. Scoring criteria: As for the scoring system of the CD-RISC, it contains 25 items, all of which carry a 5-point range of responses, as follows: not true at all (0), rarely true (1), sometimes true (2), often true (3) and true nearly all the time (4). The scale is rated based on how the subject has felt over the past month. The total score ranges from 0 to 100, with higher scores reflecting greater resilience. Cronbach’s alpha coefficient for the full scale was 0.89 [31].

#### Instrument III Perceived Stress Scale (PSS-10)

The Perceived Stress Scale (PSS-10) was developed by Cohen and colleagues, (1983) [32]. Perceived Stress Scale (PSS) is widely considered the gold standard instrument for measuring stress perception. This questionnaire is used to assess the degree to which a respondent finds circumstances in their life to be unpredictable, uncontrollable and/or overwhelming. Questions on the PSS ask the respondent to rate their feelings and thoughts for the past month, indicating that a relatively recent time frame is assessed. PSS was designed to measure the degree to which situations in one’s life are appraised as stressful. This version of the PPS had 10 items: four positive items (items 4, 5, 7, 8) and six negative items (items 1, 2, 3, 6, 9 and 10). Each item is scored on a 5-point scale, ranging from 0 (never) to 4 (very often). The positive elements’ ratings are reversed, for example, 0 = 4, 1 = 3, 2 = 2, etc. and then summing across all 10 items, a higher score indicates more perceived stress. The internal consistency of the questionnaire with Cronbach’s alpha, was 0.866 [33].

#### Instrument IV Sexual satisfaction scale

The Sexual Satisfaction scale which was developed by Štulhofer and colleagues, (2010) [34]. It contained 20 items divided into two subscales as follows: a) Ego focused on 10 items and Partner and activity focused 10 items. Every item is scored on a 5-point rating system: 1 = not at all satisfied. 2 = a little satisfied. 3 = moderately satisfied. 4 = very satisfied. 5 = extremely satisfied. Estimates of internal reliability for the produced acceptable Cronbach’s alpha coefficients to the full scale 0.94 [34].

### Procedures

A pilot study, or preliminary investigation, was carried out on a randomly chosen cohort of 10 participants constituting 10% of the total women under examination. Before the commencement of the primary research study, the primary objectives of this pilot study were to evaluate the clarity, applicability and practicality of the study instruments, measure the time required for participants to complete the instruments and identify any potential challenges anticipated during data collection. The outcomes of the pilot study indicated that no modifications were deemed necessary; the instruments exhibited clarity and vibrancy. Notably, participants involved in the pilot study were deliberately excluded from the overall sample of the research to ensure result consistency.

The data collection for this study was instituted after obtaining ethical approval from faculty of Medicine Beni-Suef University followed by the approval from the hospital authorities to conduct the current study after an explanation of the aim of the study. For this study, a well-structured interview using a previously prepared questionnaire with the participants using the participants’ mother tongue (Arabic) was employed. The researchers used the questionnaire, incorporating relevant Connor-Davidson Resilience scale items, Perceived Stress Scale and the Sexual Satisfaction Scale from related studies. The instruments were translated from English to Arabic at the Center for Specialized Languages, Faculty of Arts, Helwan University. A panel of experts in psychology, psychiatry and psychiatric nursing then evaluated the instrument’s validity and suitability for use with mastectomized women. Then the questionnaire was distributed by the researchers through performing interviews with the participants in the outpatient clinics, Beni-Suef University Hospital. Researchers measured the reliability for Perceived Stress Scale = 0.92, Resilience scale = 0.86 and the Sexual Satisfaction Scale = 0.93.

The survey was successfully conducted with participants in the hospital in the form of well-structured interviews, where the researchers meet the participants, explained the aim of the study to them, assessed their ability to participate, obtained their written informed consent; the informed consent, explaining the study’s aim, confidentiality guarantees and the ability to withdraw from the study at any time without any fear. Then the data was collected. The Emotional freedom technique was applied through various phases. Preparatory phase involved reviewing the studies and available literature related to the research problem and theoretical knowledge using evidence-based articles, textbooks, internet periodicals and journals. The second phase, the assessment phase, involved daily communication between the researchers and the nursing supervisors in the outpatient department. During this phase, the researchers gathered information on the number of patients recently admitted to the surgical department and verified if they met the inclusion criteria. Additionally, the researchers inquired about the patients’ scheduled follow-up visits in the outpatient clinic. The researchers interviewed the participants (pre- the emotional freedom technique interventions) to complete the socio-demographic, perceived Stress, resilience and sexual Satisfaction. The interview lasted between 25 and 35 min approximately.

Planning Phase (development of nursing application of emotional freedom technique): The objectives were formulated with careful consideration of the needs of the women involved, drawing on recent significant research and expert validation from the nursing field. These objectives were revised and refined based on expert feedback to ensure they could be applied in various ways. One such approach included the creation of a booklet, which highlighted the key aspects of the emotional freedom technique specifically for women who had undergone mastectomies. The booklet, designed by researchers, was written in simple Arabic and accompanied by illustrative photos. Additionally, the researchers, who are faculty members in the College of Nursing, began by learning how to practice the technique themselves and completed specialized training courses to become proficient in training others.

The implementation of the EFT interventions was carried out by a team of well-trained researchers with specialized knowledge in EFT and its application in stress management in a classroom-like room at the outpatient clinic, which had been specially prepared by the hospital’s training department to be suitable for training. The sessions, which included 8–9 participants each, were structured to incorporate a series of lectures, brainstorming activities, discussions and simulations, supplemented by photos, illustrations and graphs. The total of 112 participants were divided into smaller groups to facilitate more personalized interaction and practice. This interactive, classroom-like setting enabled the mothers to engage actively and practice the techniques in a supportive and controlled environment.

The intervention consisted of six structured EFT sessions delivered over six weeks, with each participant receiving one session per week, totaling six sessions per participant. To accommodate participants’ schedules and ensure their continued engagement, sessions were conducted three times per week for different groups, allowing flexibility in attendance. The session schedules were coordinated with the follow-up appointments at the outpatient clinic, ensuring that participants did not have to make additional visits solely for the sessions. This approach minimized their burden and enhanced their commitment to the program.

Each session was structured into two parts: the first 25 min provided theoretical information about breast cancer, including its symptoms, treatment options, and psychological effects on stress, resilience, and sexual satisfaction. The second part, lasting 30 min, involved a step-by-step demonstration and guided practice of the Emotional Freedom Technique. Participants were thoroughly guided through both the theoretical and practical components to ensure a comprehensive understanding of the technique’s benefits. The evaluation phase assessed participants’ perceived stress, resilience, and sexual satisfaction before and after the intervention using the same tools, enabling a precise comparison of pre- and post-intervention outcomes.

### Statistical analysis

Data analysis was performed using SPSS 26.0 (IBM Inc., Chicago, IL, USA) to examine the survey responses from the recruited women. Descriptive statistics, including frequencies (percentages) and mean ± standard deviations (SD), were utilized to summarize both the value general characteristics of the participants and the scores obtained on various scales. The normality of the data was assessed using the Shapiro-Wilk test and visual inspection of histograms and Q-Q plots. Paired t-tests for comparing the mean scores between two periods within the same group. Correlation between different numerical variables was tested using Pearson product-moment correlation coefficient and spearman correlation for categorical variables. Probability (p-) less than 0.05 was considered significant and less than 0.001 considered as highly significant).

## Results

Table 1 summarizes the demographic and medical characteristics of the study participants. The majority (31.3%) were aged between 40 and 49 years, with 52.7% being married and 50.9% identifying as housewives. In terms of literacy, 32.1% were able to read and write. Geographically, 36.3% resided in rural areas and 70.5% reported having an inadequate monthly income. From a medical perspective, 42% of participants had been diagnosed with breast cancer for less than one year and 70.5% had a family history of breast cancer. Additionally, 42% had undergone a mastectomy within the past week and 51.8% did not have any chronic diseases (Table 1).

**Table 1:** Participant characteristics at baseline (*n* = 112)

Personal/Medical data	No	%
**Age**		
• 20 to < 30	23	20.5
• 30 to < 40	22	19.6
• 40 to < 50	35	31.3
• ≥50	32	28.6
**Occupation**		
• Working	55	49.1
• Housewife	57	50.9
**Educational level**		
• Illiterate	21	18.8
• Literate	36	32.1
• Secondary Education	22	19.6
• University Education	22	19.6
• Post Graduate Education	11	9.8
**Place of Residence**		
• Rural	63	56.3
• Urban	49	43.8
**Income status**		
• Enough	33	29.5
• Not enough	79	70.5
**Medical history**		
**Breast cancer duration**		
• Less than one year	47	42.0
• 1 < 5 years	31	27.7
• ≥ 5 years	34	30.4
**Family Members with Breast Cancer**		
• Yes	79	70.5
• No	33	29.5
**Mastectomy duration**		
• 1 week	47	42
• 2 weeks	10	8.9
• 3 weeks	34	30.4
• 1 month	21	12.7

Our study findings demonstrate statistically significant differences between pre- and post-intervention for all study variables. The total mean score for perceived stress decreased from 32.42 ± 1.696 at pre-intervention to 17.27 ± 2.962 at post-intervention (t = 49.130, *P* < 0.001) with effect sizes = 5.785 and CI= (14.5406–15.762). For resilience, the mean score increased from 11.53 ± 1.670 at pre-intervention to 31.46 ± 5.477 at post-intervention (t = 36.454, *P* < 0.001) with effect sizes = 3.2638 and CI= (21.011–18.845). Additionally, sexual satisfaction showed a significant increase, with the mean score rising from 17.03 ± 1.553 at pre-intervention to 31.0 ± 4.309 at post-intervention (t = 13.245, *P* < 0.001) with effect sizes = 3.8891and CI= (14.701–13.245). These results underscore the significant improvements in perceived stress, resilience and sexual satisfaction among participants following the intervention (Table 2).

**Table 2:** Pre- and Post-Intervention changes in perceived stress, resilience, and sexual satisfaction (*n* = 112)

Study variables	Time point	M	SD	t	p	ES* (Cohen’s d)	SE	df	CI	
									Lower	Upper
Perceived Stress	T0	32.4286	1.69639	49.130	< 0.000	5.785	0.30840	11	14.540	15.762
	T1	17.2768	2.96286							
Resilience	T0	11.5357	1.67078	36.454	< 0.000	3.2638	0.54668	11	21.011	18.845
	T1	31.4643	5.47711							
Sexual Satisfaction	T0	17.0357	1.55342	38.023	< 0.000	3.8891	0.36749	11	14.701	13.245
	T1	31.0089	4.30900							

T0, baseline; T1, Post intervention; M, Mean; SD, Standard Deviation; SE = Std. Error, CI = 95% Confidence Interval; df, degree of freedom

Findings of the current study Show that there was high statistically significant positive correlation between the participants’ sexual satisfaction and resilience at post program at (*r* = 0.890 & *P* < 0.001), while there was high Statistically significant negative correlation between perceived stress and participants resilience at (*r*=-0.692 & *P* < 0.01) and between perceived stress at sexual satisfaction at (*r*=-0.835 & *P* < 0.01) (Table 3).

**Table 3:** Correlations between study variables among participants patients post intervention (*n* = 112)

Variables		Perceived Stress	Resilience	Sexual Satisfaction
Perceived Stress	r P	1		
Resilience	r P	− 0.692** 0.000	1	
Sexual Satisfaction	r P	− 0.835** 0.000	0.890** 0.000	1
α		0.923	0.871	0.881

r = Pearson Correlation, **. Correlation is highly significant at the 0.01 level (2-tailed), α = Cronbach’s Alpha

**Table 4:** Multilinear regression model to analysis effect of patients characteristics on study variables over post intervention (*n* = 112)

Model	Model 1 (resilience)			t	*P*	Model 2 (stress)			t	Sig.	Model 3 (sexual satisfaction)			t	*P*
	B	SE	Beta			B	SE	Beta			B	SE	Beta		
(Constant)	53.822	1.926		27.939	0.000	6.624	3.198		2.071	0.041	28.876	3.028		9.535	0.000
Age	-4.294-	0.241	− 0.863-	-17.785	0.000**	1.072	0.401	0.398	2.674	0.009*	-1.481-	0.380	− 0.378-	-3.901-	0.000**
Occupation	− 0.560-	0.564	− 0.051-	− 0.994	0.323	0.228	0.936	0.039	0.244	0.808	7.945	0.886	0.926	8.963	0.000**
Educational level	− 0.854-	0.247	− 0.196-	-3.462-	0.001**	0.259	0.409	0.110	0.632	0.529	2.915	0.388	0.851	7.519	0.000**
Residence	− 0.704-	0.304	− 0.064-	-2.315-	0.023*	0.730	0.505	0.123	1.446	0.151	-3.203-	0.478	− 0.370-	-6.699-	0.000**
Income	-5.189-	0.593	− 0.434-	-8.749-	0.000**	0.540	0.985	0.084	0.549	0.584	-6.039-	0.932	− 0.642-	-6.477-	0.000**
F R R^2^			811.143** 0.989 0.979					70.477** 0.895 0.801					190.026** 0.957 0.916		

B, Unstandardized Coefficients; SE, Std. Error; Beta, Standardized Coefficients; F, ANOVA Test; R^2^, R Square

Findings of our study Clarify the effect of participants’ patients characteristics on the study variables through the post program phase, model 1 illustrates that there was statistically significant effect of age, educational level and monthly income on their resilience variable at (*P* < 0.05). Regarding model 2 there was statistically significant effect of participants’ patients age on perceived stress variable at (*P* < 0.05). Concerning Model 3, there was statistically significant effect of the participants’ patients age, job, educational level, residence and monthly income on their sexual satisfaction at (*P* < 0.05) (Table 4).

## Discussion

Regardless of the prognosis, a breast cancer diagnosis still causes women more distress than any other medical diagnosis, despite advancements in early detection and treatment [35]. This anguish can take many different forms, from symptoms of post-traumatic stress disorder to psychiatric illness like anxiety or depression [36]. From the time of diagnosis until the end of life, patients with breast cancer may feel stress. It was anticipated that at the moment of diagnosis, there would be a greater degree of psychological suffering affecting resilience and sexual satisfaction. This study was the first study concerned with the effect of EFT on perceived stress, resilience and sexual satisfaction among women after mastectomy.

In the current study, the prevalence of perceived stress, resilience and sexual satisfaction was 32.42 ± 1.696, 11.53 ± 1.670 and 17.03 ± 1.553 respectively. Regardless different the studied variables, the results of the present study were in line with the study by Afriyanti and Wenni, (2018) reported that the self-concept of women with breast cancer after mastectomy was carried out, the majority had negative self-concept, namely 29 respondents (87.9%) [10]. According to Ghaderi and colleagues, (2021) when a woman is diagnosed with breast cancer, not only has an impact on her physical but also on her emotional and mental state, which can then affect her relationship with others. They begin to be alone and the response to rejection of the truth of the diagnosis continues to occur [37]. Another study done by Alagizy and colleagues, (2020) agreed with the present study findings reported that the prevalence of stress in the study group was 78.1%. The mean score of Perceived Stress Scale (PSS) was 17.89 ± 7.55. Fourteen women (21.9%) had low stress level, 44 (68.8%) had moderate and 6 women (9.4%) had high stress level [12].

A comparison of pre- and post-intervention results revealed a significant improvement in the mean scores of women’s perceived stress, resilience, and sexual satisfaction following the implementation of EFT. These findings indicate that EFT had a substantial impact on the study variables, as evidenced by a reduction in perceived stress from 32.42 ± 1.70 to 17.27 ± 2.96. Additionally, resilience scores increased significantly from 11.53 ± 1.67 to 31.46 ± 5.48 after the intervention. This result aligns with the findings of Ghamsari and Lavasani (2015), who reported that EFT effectively reduces perceived stress and enhances resilience in participants [20]. Conversely, Ahn and Suh (2023) highlighted that some women experienced increased strength and self-assurance following mastectomy, rejecting conventional beauty standards and embracing their scars with a sense of pride [38]. This perspective suggests that post-mastectomy experiences and coping mechanisms can vary, with some women finding empowerment in their journey while others benefit from structured interventions like EFT to manage psychological distress.

The effectiveness of EFT in reducing perceived stress can be understood through its integration of cognitive and somatic processes. According to the Transactional Model of Stress and Coping, stress arises when individuals perceive environmental demands to exceed their coping resources [39, 40]. EFT interrupts this cycle by combining cognitive awareness of stressors with the physiological act of tapping on acupressure points, which helps to regulate stress responses [41]. This dual approach reduces hyperarousal in the body by calming the autonomic nervous system, potentially lowering cortisol levels and facilitating relaxation. Furthermore, by addressing distressing thoughts during tapping, EFT allows for cognitive reframing, helping individuals reappraise stressors as manageable. These mechanisms align with the study’s findings, demonstrating a significant reduction in participants’ perceived stress post-intervention.

Resilience, defined as the ability to recover from adversity, is influenced by both emotional regulation and adaptive coping strategies [42]. EFT contributes to resilience by enabling individuals to process and release negative emotions effectively. As shown in this study, participants exhibited a substantial increase in resilience scores following the intervention. This can be attributed to EFT’s role in promoting emotional regulation, which reduces the impact of stressors and enhances coping capacity. Additionally, EFT fosters a sense of empowerment and self-efficacy by equipping individuals with a tool to address their emotional challenges. Regular practice of EFT has been shown to strengthen resilience over time, enabling individuals to maintain emotional stability and engage in positive health behaviors [43]. These findings highlight the potential of EFT to build resilience, particularly in vulnerable populations such as mastectomized women.

Findings of the current study revealed an increase of sexual satisfaction from 17.03 ± 1.553 to 31.0 ± 4.309 post technique implementation. This finding agreed with the finding of the Egyptian study conducted by Abd Elhafeez and Colleagues, (2024) who reported that Sexual Counseling improved the sexual Pleasure and Satisfaction among mastectomized women [22]. Sexual satisfaction is intricately linked to emotional and psychological well-being, both of which are addressed by EFT. The Biopsychosocial Model of Sexual Health underscores the interrelationship between psychological distress, body image and sexual satisfaction [44, 45] In this study, EFT significantly improved participants’ sexual satisfaction, likely by reducing stress and anxiety, which are known inhibitors of sexual desire and performance. By alleviating negative body image and enhancing emotional stability, EFT allows women to feel more confident and connected in their intimate relationships. Furthermore, the reduction in perceived stress through EFT may improve physiological responses to intimacy, such as arousal and lubrication, thereby enhancing overall sexual satisfaction. These findings suggest that EFT can serve as a holistic intervention to address the multifaceted challenges of sexual health among women post-mastectomy.

Our findings provide new evidence for identifying the correlation between the participants’ perceived stress and resilience at post program. This finding is in line with the study conducted by Wimberly and colleagues, (2005) who supported the idea of positive emotions not only indicate recovery but also influence health and speed up the recovery [46]. Fredrickson and colleagues, (2003) agreed with our study findings when they reported in their study that people in crisis have shown that activation of positive emotions (such as love, hope, or gratitude) in the context of negative emotions may be crucial for resilience [47]. Also, our study finding was consistent with Garcia-Leon and colleague, (2019) whose study reported that resilience was associated with chronic stress by perceived stress (*p* < 0.001) [48]. However, there is no consensus about if resilience is related to the different perceptual, self-report and cortisol-based measures of stress such as daily or chronic stress and psychopathology. While the study finding proved a significant positive correlation between sexual satisfaction and participants resilience. Resilience indicates the capacity to overcome stressful situations, such as mastectomy.

Our study findings were in the same line of the study findings of Oliva and colleagues, (2022) who found sexual function was significantly worse in the low resilience subgroup of participants, with an FSFI total score of 18.90 [14.10–24] versus 29.40 [24.60–33] in the mid-high resilience one (*P* < 0.001). Ultimately, the finding of our study denoted that there was a negative correlation between perceived stress at sexual satisfaction [49]. Also, the study of Abedi and colleagues, (2014) was in the same line demonstrating that there was a significant correlation between stress and sexual satisfaction (*p* < 0.001) [50].

Our study result clarified the effect of participants’ characteristics on the study variables post EFT implementation, there was statistically significant effect of age on perceived stress variable. This result was disagreed with Li and colleagues, (2021), who reported that sociodemographic characteristics, including years of education, occupation, residence, monthly household income and religion were associated with perceived stress (PS < 0.1) and this indicated the mediating role of perceived stress [51]. Also the finding findings illustrated that there was statistically significant effect of participants’ age, educational level and monthly income on resilience variable. This finding is in the same line of the study conducted by Ostadi-Sefidan and colleagues, (2024) who studied resilience and its related factors among women with breast cancer and showed that social support, hope, age and income level predict significant resilience in women with breast cancer [52]. The present study result revealed that there was statistically significant effect of the participants’ patients age, job, educational level, residence and monthly income on sexual satisfaction. This result was contradicted by study performed by Hamed and colleagues, (2015) who pointed out that there was no statistically significant relation between quality of sexual life and education, the high education had the high quality of sexual life scores with mean of (43.85) [53].

## Conclusion

This study underscores the EFT as an effective, non-invasive intervention for reducing stress, enhancing resilience and improving sexual satisfaction among women post-mastectomy. To support its integration into nursing practice, structured in-service training programs should be implemented, incorporating theoretical foundations, hands-on practice and competency assessments. Standardizing EFT within post-mastectomy nursing protocols and providing continuous mentorship can further enhance its application. At the policy level, integrating EFT into oncology care guidelines and hospital rehabilitation programs can ensure sustainability. Future research should explore its long-term benefits, applicability across diverse settings and potential for telehealth delivery. By adopting these strategies, EFT can become a valuable, cost-effective component of post-mastectomy care, improving recovery outcomes and quality of life.

### Implication

The findings of this study highlight the Emotional Freedom Technique as an effective, non-invasive intervention for reducing stress, enhancing resilience and improving sexual satisfaction in women post-mastectomy. Given the observed correlation between perceived stress and resilience post-program, integrating EFT into clinical practice can enhance patient care through structured implementation. To achieve this, nurses should receive standardized training and certification in EFT, ensuring competency in its application. Establishing evidence-based clinical protocols, including session frequency, duration and assessment measures, can facilitate its integration into post-mastectomy rehabilitation and oncology follow-ups.

Additionally, patient education should be prioritized by providing structured materials, such as brochures, instructional videos and mobile applications, to promote self-practice and adherence. Multidisciplinary collaboration among oncology nurses, psychologists and physiotherapists is essential to incorporating EFT into holistic recovery plans. Furthermore, resource allocation and policy advocacy can support the recognition of EFT as a complementary therapy within oncology care guidelines, increasing accessibility in hospital and community healthcare settings. By implementing these structured strategies, EFT can become a valuable component of patient-centered post-mastectomy care, empowering women in their recovery journey and improving their overall quality of life.

### Limitation of the study

This study offers valuable insights into the effectiveness of the nursing application of EFT for reducing stress, enhancing sexual satisfaction and improving the psychological well-being of mastectomized women, but several limitations must be acknowledged. First, the study used a convenience sample of 112 Egyptian mastectomized women, which limits the generalizability of the findings to broader populations. The sample’s homogeneity in terms of geographic location and cultural context may not represent the diversity of women with breast cancer globally and future research with more diverse, multi-country samples is necessary. The quasi-experimental design, while informative, limits the ability to draw definitive conclusions about causality and the absence of a randomized controlled trial (RCT) reduces the strength of evidence regarding the effectiveness of EFT. Additionally, the study’s short-term follow-up period, with outcomes assessed immediately post-intervention, leaves the sustainability of effects over time unclear. Longitudinal studies with extended follow-up would provide more clarity. The reliance on self-reported measures introduces potential biases such as social desirability or recall bias and future studies could benefit from incorporating objective assessments. Furthermore, cultural and contextual factors in Egypt, such as local healthcare practices and attitudes toward cancer, may influence psychological responses, suggesting the need for research that accounts for cultural variations in EFT’s effectiveness.

### Ethical consideration

Approval by the Research Ethics Committee (REC) was utilized and the college of Medicine, Beni-Suef University, Egypt provided ethical permission prior to the study’s conduct (Approval No: FMBSUREC/03122023/Mohamed). Prior to taking part in the study, participants had to give written informed consent as proof of their voluntary participation. Strict procedures were followed throughout the study to protect the participants’ privacy. All personally identifiable information was kept private and available only to the research team. It was crucial to protect the participants’ confidentiality and privacy. All procedures of this study were conducted in accordance with the ethical principles outlined in the Declaration of Helsinki and its subsequent amendments. Additionally, the study is registered at ClinicalTrials.gov under the registration number NCT06583629 on 4/9/2024.

## Data Availability

The corresponding author can provide the datasets used and/or analyzed for this study upon reasonable request.

## Abbreviations

EFT: Emotional Freedom Technique
PSS: Perceived Stress Scale
CD-RISC: Connor-Davidson Resilience Scale

## References

[1] Nejad SB, Kargar A, Hamid N, Razmjoo S. Metacognitive beliefs, positive States of Mind, and emotional approach coping as the predictors of medical compliance in patients with cancer. Int J Cancer Manag. 2020;13(7):1–6.

[2] World Health Organization. Breast cancer. Retrieved from https://www.who.int/news-room/fact-sheets/detail/breast-cancer. 2023;(March):1–6.

[3] Giaquinto AN, Sung H, Newman LA, Freedman RA, Smith RA, Star J et al. Breast cancer statistics 2024. CA Cancer J Clin [Internet]. 2024:477–95. Available from: http://www.ncbi.nlm.nih.gov/pubmed/39352042 10.3322/caac.21863 39352042

[4] Azamjah N, Soltan-Zadeh Y, Zayeri F. Global trend of breast cancer mortality rate: A 25-year study. Asian Pac J Cancer Prev. 2019;20(7):2015–20. 31350959 10.31557/APJCP.2019.20.7.2015 PMC6745227

[5] World Health Organization/International Agency for Research on Cancer. [Internet]. 2020. Available from: https://gco.iarc.fr/today/data/factsheets/populations/818-egypt-fact-sheets.pdf. Accessed September 2023.

[6] Ministry of Health and Family Welfare. National Multisectoral Action Plan for Prevention and Control of Common Noncommunicable Diseases. 2017.

[7] Mahmoud MR, Alharbi DSB, Alhammad RAH, Hegazy ME, Abdelaziz OG, Abdelaziz SG. Comparative assessment of breast cancer prevalence between women in Saudi Arabia and Egypt. Med Sci. 2023;27(135):1–12.

[8] Li X, Zhang X, Liu J, Shen Y. Prognostic factors and survival according to tumour subtype in women presenting with breast cancer bone metastases at initial diagnosis: a SEER-based study. BMC Cancer. 2020;20(1):1–12. 10.1186/s12885-020-07593-8 PMC7666499 33187507

[9] Sangwan RK, Huda RK, Panigrahi A, Toteja GS, Sharma AK, Thakor M, et al. Strengthening breast cancer screening program through health education of women and capacity Building of primary healthcare providers. Front Public Heal. 2023;11(November):1–10. 10.3389/fpubh.2023.1276853 PMC10687205 38035296

[10] Afriyanti E, Wenni BP. The effect of spiritual emotional freedom technique (SEFT) on the self concept of breast cancer patients with mastectomy. J Keperawatan Padjadjaran. 2018;6(3):243–52.

[11] Kocan S, Gursoy A. Body image of women with breast cancer after mastectomy: A qualitative research. J Breast Heal. 2016;12(4):145–50. 10.5152/tjbh.2016.2913 PMC5351438 28331752

[12] Alagizy HA, Soltan MR, Soliman SS, Hegazy NN, Gohar SF. Anxiety, depression and perceived stress among breast cancer patients: single institute experience. Middle East Curr Psychiatry. 2020;27(1).

[13] Nooripour R, Ghanbari N, Hosseinian S, Hassani-Abharian P, Dobkins K, Maadal A. Effectiveness of mindfulness-based cognitive rehabilitation in reducing stress among hard of hearing adolescent girls. Int J Behav Sci. 2021;15(2):87–93. 10.30491/ijbs.2021.253241.1400

[14] Aizpurua-Perez I, Perez-Tejada J. Resilience in women with breast cancer: A systematic review. Eur J Oncol Nurs [Internet]. 2020;49:101854. Available from: 10.1016/j.ejon.2020.101854 10.1016/j.ejon.2020.101854 33120216

[15] Brajkovic L, Sladic P, Kopilaš V. Sexual quality of life in women with breast cancer. Heal Psychol Res. 2021;9(1):1–14. 10.52965/001c.24512 PMC8567769 34746481

[16] Relationship between hardiness and stress of COVID-19 through the mediating role of mindfulness in Iranian students. Pract Clin Psychol. 2022;10(3):193–202.

[17] Desoky M, Abdo Hussien A, Ibrahim A, Metwally H. Emotional freedom technique for reducing primary dysmenorrhea intensity among female students. Assiut Sci Nurs J. 2023;11(37):33–42.

[18] Balha M, Abo-Baker S, Mahmoud O. Effect of emotional freedom techniques on psychological symptoms and cravings among patients with substance related disorders. Int J Nov Res Healthc Nurs [Internet]. 2020;7(2):30–45. Available from: www.noveltyjournals.com.

[19] Feinstein D. Acupoint stimulation in treating psychological disorders: evidence of efficacy. Rev Gen Psychol. 2012;16(4):364–80.

[20] Ghamsari MsarvandiL. Effectiveness of emotion freedom technique on pregnant women’s perceived stress and resilience. J Educ Sociol. 2015;6(April):118–22.

[21] Ashton K, Oney K. Psychological intervention and breast cancer. Curr Breast Cancer Rep [Internet]. 2024;311–9. Available from: 10.1007/s12609-024-00559-w

[22] Abdelhafez M, Abdullah AO, Fathy Ahmed S, Badia NS. Effect of group counseling based on Problem-Solving solution on women’s sexual function, quality of life and sexual satisfaction after mastectomy. Egypt J Heal Care. 2024;15(1):2028–39.

[23] Borgi M, Collacchi B, Ortona E, Cirulli F. Stress and coping in women with breast cancer:unravelling the mechanisms to improve resilience. Neurosci Biobehav Rev. 2020;119(October):406–21. 33086128 10.1016/j.neubiorev.2020.10.011

[24] Lazarus RS, Folkman S. Transactional theory and research on emotions and coping. Eur J Pers. 1987;1(3):141–69.

[25] Lekeka M. Psychosocial group intervention at a Low-Resource setting environment for women who are diagnosed and treated for breast cancer: A systematic review. Health (Irvine Calif). 2023;15(10):1150–70.

[26] Singtaweesuk N, Thanoi W, Vongsirimas N, Kesornsri S, Klainin-Yobas P. Factors predicting psychological Well-being among survivors of breast cancer in A tertiary care hospital, Thailand. Siriraj Med J. 2024;76(5):244–54.

[27] Vegunta S, Kuhle CL, Vencill JA, Lucas PH, Mussallem DM. Sexual health after a breast cancer diagnosis: addressing a forgotten aspect of survivorship. J Clin Med. 2022;11:22. 10.3390/jcm11226723 PMC9698007 36431200

[28] Cherven BO, Demedis J, Frederick NN. Sexual health in adolescents and young adults with cancer. J Clin Oncol. 2024;42(6):717–24. 37856773 10.1200/JCO.23.01390

[29] Dewi EU, Nursalam, Mahmudah, Yunitasari E. The effect of peer support psychoeducation based on experiential learning on self-care demands among breast cancer patients with post-chemotherapy. J Public Health Res. 2023;12(1). 10.1177/22799036221146901 PMC9834624 36643605

[30] Kang H. Sample size determination and power analysis using the G*Power software. J Educ Eval Health Prof. 2021;18:1–12. 34325496 10.3352/jeehp.2021.18.17 PMC8441096

[31] Connor KM, Davidson JRT. Development of a new resilience scale: the Connor-Davidson resilience scale (CD-RISC). Depress Anxiety. 2003;18(2):76–82. 12964174 10.1002/da.10113

[32] Cohen S, Kamarck T, Mermelstein R. A global measure of perceived stress. J Health Social Behav J Health Soc Behav. 1983;24(4):385–96. 6668417

[33] Amaral AP, Soares MJ, Bos SC, Pereira AT, Marques M, Valente J, et al. The perceived stress scale (PSS-10)-a Portuguese version. Clínica. 1991;12:187–93.

[34] Štulhofer A, Buško V, Brouillard P. Development and bicultural validation of the new sexual satisfaction scale. J Sex Res. 2010;47(4):257–68. 19629836 10.1080/00224490903100561

[35] Shapiro SL, Lopez AM, Schwartz GE, Bootzin R, Figueredo AJ, Braden CJ, et al. Quality of life and breast cancer: relationship to psychosocial variables. J Clin Psychol. 2001;57(4):501–19. 11255204 10.1002/jclp.1026

[36] Meunier J, Libert Y, Delvaux N, Marchal S, Etienne A, Lienard A, et al. Psychobiological correlates of communication skills use and learning: preliminary results. Psycho-Oncology J Psychol Soc Behav Dimens cancer. 2007;16(9):181–8.

[37] Ghaderi Z, Nazari F, Shaygannejad V. The effect of emotional freedom technique on fatigue among women with multiple sclerosis: A randomized controlled trial. Iran J Nurs Midwifery Res. 2021;26(6):531–6. 34900653 10.4103/ijnmr.IJNMR_188_19 PMC8607895

[38] Ahn J, Suh EE. Body image alteration in women with breast cancer: a concept analysis using an evolutionary method. Asia-Pacific J Oncol Nurs [Internet]. 2023;10(5):100214. Available from: 10.1016/j.apjon.2023.100214 10.1016/j.apjon.2023.100214 PMC10199402 37213808

[39] Lazarus R, Folkman S. Stress, appraisal, and coping. Springer; 1984;464.

[40] Ryu G. A theoretical integration of work–family studies with the transactional model of stress. J Fam Theory Rev. 2024;16(2).

[41] Bougea AM, Spandideas N, Alexopoulos EC, Thomaides T, Chrousos GP, Darviri C. Effect of the emotional freedom technique on perceived stress, quality of life, and cortisol salivary levels in tension-type headache sufferers: A randomized controlled trial. Explor J Sci Heal [Internet]. 2013;9(2):91–9. Available from: 10.1016/j.explore.2012.12.005 10.1016/j.explore.2012.12.005 23452711

[42] Sisto A, Vicinanza F, Campanozzi LL, Ricci G, Tartaglini D, Tambone V. Towards a transversal definition of psychological resilience: A literature review. Med. 2019;55(11):1–22. 10.3390/medicina55110745 PMC6915594 31744109

[43] Spengler PM, Lee NA, Wiebe SA, Wittenborn AK. A comprehensive Meta-Analysis on the efficacy of emotionally focused couple therapy. Couple Fam Psychol Res Pract. 2022;13(2):81–99.

[44] Berry MD, Berry PD. Contemporary treatment of sexual dysfunction: reexamining the biopsychosocial model. J Sex Med. 2013;10(11):2627–43. 23937720 10.1111/jsm.12273

[45] Rullo J, Faubion SS, Hartzell R, Goldstein S, Cohen D, Frohmader K et al. Biopsychosocial management of female sexual dysfunction: a pilot study of patient perceptions from 2 multi-disciplinary clinics. Sex Med [Internet]. 2018;6(3):217–23. Available from: 10.1016/j.esxm.2018.04.003 10.1016/j.esxm.2018.04.003 PMC6085226 29789244

[46] Wimberly SR, Carver CS, Laurenceau JP, Harris SD, Antoni MH. Perceived partner reactions to diagnosis and treatment of breast cancer: impact on psychosocial and psychosexual adjustment. J Consult Clin Psychol. 2005;73(2):300–11. 15796638 10.1037/0022-006X.73.2.300

[47] Fredrickson BL, Tugade MM, Waugh CE, Larkin GR. What good are positive emotions in crisis? J Pers Soc Psychol. 2003;84(2):365–76. 12585810 10.1037//0022-3514.84.2.365 PMC2755263

[48] García-León MÁ, Pérez-Mármol JM, Gonzalez-Pérez R, García-Ríos M, del Peralta-Ramírez C. MI. Relationship between resilience and stress: Perceived stress, stressful life events, HPA axis response during a stressful task and hair cortisol. Physiol Behav [Internet]. 2019;202:87–93. Available from: 10.1016/j.physbeh.2019.02.001 10.1016/j.physbeh.2019.02.001 30726720

[49] Oliva A, Serrano-García I, Asenjo JE, Cedeira E, Gil-Prados I, Herraiz MA, et al. Resilience and sexual health among menopausal women: A cross-sectional study. Menopause. 2022;29(4):408–14. 35357364 10.1097/GME.0000000000001935

[50] Abedi P, Afrazeh M, Javadifar N, Saki A. The relation between stress and sexual function and satisfaction in reproductive-Age women in Iran: a cross-sectional study. J Sex Marital Ther [Internet]. 2014;0(0):1–7. Available from: 10.1080/0092623X.2014.915906 10.1080/0092623X.2014.915906 24884353

[51] Li J, Gao W, Yang Q, Cao F. Perceived stress, anxiety, and depression in treatment-naïve women with breast cancer: a case-control study. Psychooncology. 2021;30(2):231–9. 32969126 10.1002/pon.5555

[52] Ostadi-Sefidan H, Faroughi F, Fathnezhad-Kazemi A. Resilience and its related factors among women with breast cancer. Eur J Cancer Prev. 2024;33(2):129–35. 37702615 10.1097/CEJ.0000000000000839

[53] Hamed S, Mahgoub N, Esmail M, El-etreby R. Quality of sexual life among post mastectomy women. Mansoura Nurs J. 2015;2(2):23–33.

